# Immune regulator ABIN1 suppresses HIV-1 transcription by negatively regulating the ubiquitination of Tat

**DOI:** 10.1186/s12977-017-0338-5

**Published:** 2017-02-13

**Authors:** Shiyou Chen, Xiaodan Yang, Weijia Cheng, Yuhong Ma, Yafang Shang, Liu Cao, Shuliang Chen, Yu Chen, Min Wang, Deyin Guo

**Affiliations:** 1 State Key Laboratory of Virology and Modern Virology Research Center, College of Life Sciences, Wuhan University, Wuhan, 430072 People’s Republic of China; 2 Clinical Laboratory, General Hospital of the Yangtze River Shipping, Wuhan, 430010 People’s Republic of China; 3 School of Basic Medical Sciences, Wuhan University, Wuhan, 430071 People’s Republic of China; 4 School of Basic Medicine (Shenzhen), Sun Yat-sen University, Guangzhou, 510080 People’s Republic of China

**Keywords:** HIV-1, ABIN1, Tat, Transcription, Ubiquitination, HDM2

## Abstract

**Background:**

A20-binding inhibitor of NF-κB activation (ABIN1), an important immune regulator, was previously shown to be involved in HIV-1 replication. However, the reported studies done with overexpressed ABIN1 provided controversial results.

**Results:**

Here we identified ABIN1 as a suppressor of HIV-1 transcription since transient knockdown of ABIN1 led to increased HIV-1 replication both in transformed Jurkat T cell line and in primary human CD4+ T lymphocytes. Depletion of ABIN1 specifically enhanced the HIV-1 transcription from the integrated genome during viral life cycle, but not the earlier steps such as reverse transcription or integration. Immunoprecipitation assays revealed that ABIN1 specifically inhibits the proto-oncogene HDM2 catalyzed K63-linked polyubiquitination of Tat at Lys71, which is critical for the transactivation activity of Tat. The ubiquitin chain binding activity of ABIN1 carried by UBAN domain turned out to be essential for the inhibitory role of ABIN1. The results of immunofluorescence localization experiments suggested that ABIN1 may obstruct Tat ubiquitination by redistributing some of HDM2 from the nucleus to the cytoplasm.

**Conclusions:**

Our findings have revealed ABIN1 as intrinsic suppressor of HIV-1 mRNA transcription by regulating the ubiquitination of Tat.

## Background

ABIN1, a homologue of ABIN proteins, was first identified as an inhibitor of NF-κB activation. Besides ABIN1, there are two other family members ABIN2 and ABIN3 sharing limited sequence homology and each has specific functions. Their functions are non-redundant but the originally described function as NF-κB inhibitor might be redundant [1].

ABIN1 is shown to be an important regulator of the immune system, playing critical roles during the regulation of immunity as well as tissue homeostasis. Its mRNA level is especially high in immune organs and cells, such as spleen and peripheral blood leukocytes. Being essential for embryo development, ABIN1 knockout leads to embryo lethality and its dysfunction mutations cause severe abnormal development [2, 3]. With several nuclear localization signals (NLS) and a nuclear exit signal (NES) [1], ABIN1 can shuttle between nucleus and cytoplasm in the cell. In the nucleus, ABIN1 has an inhibitory role in signaling pathways to attenuate EGF/ERK2 nuclear signaling and repress nuclear receptor (NR) transcription activity [4–6]. The C-terminal bipartite ubiquitin-binding domain (UBD) of ABIN1, also named UBD in ABIN proteins and NEMO (UBAN), is homologous to NEMO ubiquitin binding domain (NUB) which binds ubiquitin chains and potentiates many functions of ABIN1, including inhibiting NF-κB signaling [7–11], preventing inflammatory and autoimmune diseases [3, 12–14] and restricting programmed cell death [2]. Recently, ABIN1 was also reported involving in macrophage M1 polarization during HCV infection and dendritic cell function [15, 16].

ABIN1 is also named as Naf1 (Nef-associated factor 1) and VAN (virion-associated matrix-interacting protein), owing to its binding ability with HIV-1 Nef and Matrix [17, 18]. Though ABIN1 was reported to be involved in HIV-1 replication, the effect of ABIN1 on HIV-1 replication was controversial. ABIN1 was shown to increase the CD4+ T cells [17] and to promote nuclear export of unspliced HIV-1 gag mRNA [19], suggesting a positive role of ABIN1 in HIV-1 replication. However, in other cases, ABIN1 has been shown to work as a shuttle protein to regulate the distribution of HIV-1 proteins whose overexpression inhibited HIV-1 replication [18], or as an inhibitor of HIV-1 LTR promoter [20], suggesting a negative role of ABIN1 in HIV-1 replication. A more thorough and detailed study need to be conducted to reveal the regulatory role of ABIN1 on HIV-1 infection, and to boost the study on the interplay between human immune system and HIV-1 infection.

HIV-1 belongs to retrovirus that specifically infects CD4+ T lymphocytes [21]. Immune system is unable to expel the virus and cure the disease [22]. Intensive studies have been done to elucidate the processes for HIV-1 infection, to seek more host inhibitory factors, and to come up with new strategies for treatment [23, 24]. Following infecting the target cells, the genome of HIV-1 was reverse transcribed and integrated into the genome of the host cell. The replication of HIV-1 depends on the active transcription of integrated HIV-1 genome driven by HIV-1 LTR promoter. The activity of LTR promoter relies on the transactivator of transcription (Tat), which binds the structured TAR elements of LTR, recruits P-TEFb complex to the transcription initial complex, and stimulates the transcription elongation [25]. The function of Tat is regulated by a variety of posttranslational modifications, including phosphorylation, methylation, acetylation and ubiquitination. HIV-1 encoded proteins, such as Vif, Gag, Vpu and Vpr have been reported intimately implicated with ubiquitination in different processes of the virus-host interactions [26–29], while only few research about the ubiquitination modification and regulation of Tat reported [30, 31].

In this paper, we constructed multiple cell lines with knockout, knockdown, or overexpressed ABIN1 to study the regulatory role of ABIN1 on HIV-1 replication. Results showed that ABIN1 was a physiologic inhibitor of HIV-1 replication by targeting the post-integration transcription step of viral replication cycle. We revealed that K63-linked polyubiquitin modification of Tat at Lys71 plays an important role during the process of Tat-stimulated HIV1 transcription, which is specifically inhibited by cellular ABIN1 protein via its polyubiquitin chain binding property. Based on our results, we also suggested that ABIN1 may inhibit Tat ubiquitination by changing the cellular distribution of the specific E3 ligase of Tat, HDM2. Thus, by confirming the inhibitory role of ABIN1 and exploring its molecular mechanisms to restrict HIV-1 mRNA transcription, our research has provided new insights in developing strategies against latent HIV-1 reservoir.

## Results

### ABIN1 knockdown increases HIV-1 replication

**Fig. 1:**
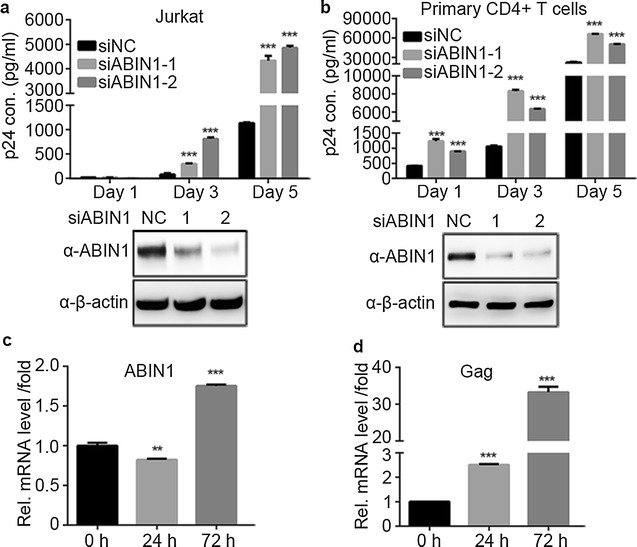
ABIN1 knockdown increases HIV-1 replication. **a** Jurkat T cells were transfected with siRNAs targeting ABIN1 (siABIN1-1 or siABIN1-2) or scramble siRNA (siNC) as described in “Methods”. 36 h post transfection (hpt), cells were counted and challenged with HIV-1 strain NL4-3. At 1, 3, 5 days post infection, the cells and supernatants were harvested to measure viral production by p24 ELISA of the supernatants, against a standard curve. Cells were lysed and subjected to Western Blots to determine the knockdown efficiency of ABIN1. The *lower panel* showed the ABIN1 knockdown efficiency at day 5 as the representative. **b** The experiments were conducted similarly as in (**a**), except that human primary CD4+ T lymphocytes were used. **c**, **d** Determination of ABIN1 mRNA level after viral infection. Jurkat cells were challenged with HIV(NL4-3) as in **a**, **b**, at 0, 24, 72 hpi, cells were harvested for RNA extraction. The mRNA levels of ABIN1 and HIV-1 gag was measured by real time PCR, normalized to cellular GAPDH. Data are shown as mean ± SD of triplicate samples and are representative of at least three independent experiments. *p* values were calculated based on unpaired *t* test and significant changes relative to siNC transfected cells or samples collected at 0 hpi. *p < 0.05; **p < 0.01; ***p < 0.001

The effect of ABIN1 on HIV-1 replication has been previously studied, but the results seem to be controversial and only cell lines such as HEK-293T or HeLa T4 cells transfected with overexpressed ABIN1 were employed during the research. To examine whether ABIN1 is able to inhibit HIV-1 replication under physiological condition, two non-overlapping siRNAs which target ABIN1 open reading frame were introduced into different cells challenged with HIV-1 (NL4-3). The results showed that transient knockdown of ABIN1 led to increased HIV-1 replication not only in Jurkat T cell line (Fig. 1a) but also in primary human CD4+ T lymphocytes, the primary target cells for HIV-1 from healthy donors (Fig. 1b).

In Jurkat T cell line, knockdown of ABIN1 by the two siRNAs significantly increased HIV-1 replication to about fourfold and tenfold at the 3rd day, and about fivefold at the 5th day post infection, measured by HIV-1 p24 (capsid) ELISA (Fig. 1a, upper panel). Western Blot analysis showed that the introduction of individual ABIN1 siRNAs into Jurkat cells resulted in around 50–80% protein level deduction at day 5 post infection (Fig. 1a, lower panel). The results suggested that ABIN1 could protect Jurkat T cell line from HIV-1 infection. When ABIN1 was depleted by siRNAs, more HIV-1 replication was observed in Jurkat T cells, especially in cells with higher knockdown efficiency and lower ABIN1 protein level (Fig. 1a).

The inhibitory role of ABIN1 on HIV-1 replication has been further confirmed by similar studies with primary CD4+ T lymphocytes from healthy donors. Activation of the lymphocytes and induction of proliferation were conducted by co-stimulation of anti-CD3 and CD28 antibodies together with IL-2, which largely mimics physiological conditions [32]. Consistent with the results obtained in Jurkat T cells, knockdown of ABIN1 increases HIV-1 replication in CD4+ T lymphocytes to twofold to threefold at day 1, sixfold to eightfold at day 3, and about threefold at day 5 post infection (Fig. 1b, upper panel). Knockdown efficiency was determined by Western Blot at day 5 post infection (Fig. 1b, lower panel). Taken together, our data reveals that the intrinsic ABIN1 in cells functions as a negative regulator to restrict HIV-1 replication in targeted host cells.

Since ABIN1 is an inhibitor of HIV-1 replication, we are curious whether HIV-1 infection would affect ABIN1 expression level during anti-viral immune response of the host cells. Jurkat T cells were challenged with HIV-1(NL4-3) and mRNA level of ABIN1 was measured by real-time PCR. The results showed that the mRNA of ABIN1 has a slight decrease at 24 h after viral infection, but afterwards increased to about twofold at 72 h post infection (Fig. 1c). The gag mRNA levels were measured to confirm HIV-1 infection during experiments (Fig. 1d). We speculated that the initial decrease of ABIN1 mRNA may be caused by the direct negative regulation of HIV-1 on antiviral regulators of host, which was then offset by later activation of host immune signaling.

In conclusion, our data suggest that ABIN1 acts as a suppressor of HIV-1 replication physiologically, and ABIN1 can be induced during anti-viral response of the host cell.

### ABIN1 depletion stimulates the activity of HIV-1 promoter

**Fig. 2:**
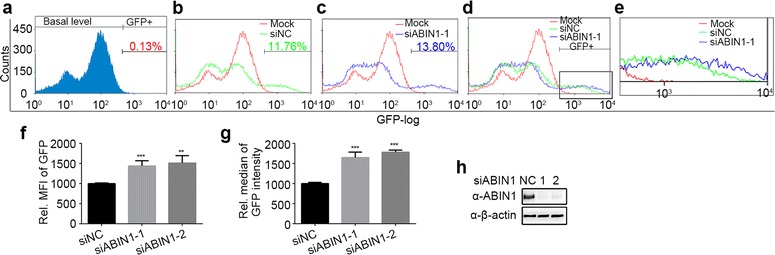
ABIN1 depletion stimulates the activity of HIV-1 promoter (Ghost-CXCR4 (X4) cells). **a**–**e** Ghost-X4 cells stably transfected a HIV-1 LTR driving GFP expression were treated with siNC, siABIN1-1, or siABIN1-2 before challenged with HIV-1(NL4-3). 48 hpi, cells were harvested and subjected to flowcytometry analysis. The effect of ABIN1 depletion on HIV-1 replication was determined by calculating the percentage of GFP positive cells, the relative mean fluorescence intensity (MFI) and relative median of GFP intensity of these GFP positive cells, over 10,000 cells were measured for each sample. **a** Indicates the basal level of GFP in non-infected Ghost-X4 cells. **b**–**d** Comparison of the cell counts at different GFP intensity after viral infection following ABIN1 knockdown. **e** The emphasis of the comparison. **f**, **g** Changing fold of MFI (**f**) and GFP intensity median (**g**) in infected cells following transfection of siRNAs targeting ABIN1 relative to siNC. The basal MFI and median of GFP intensity in siNC transfected cells were both set to 1000. **h** Representative of ABIN1 knockdown efficiency in Ghost-X4 cells. Data are shown as mean ± SD of triplicate samples and are representative of at least three independent experiments. *p* values were calculated based on unpaired *t* test and significant changes relative to siNC indicated. *p < 0.05; **p < 0.01; ***p < 0.001

To further study the role of ABIN1 on HIV-1 inhibition, we decided to determine at which stage of HIV-1 life cycle ABIN1 affects HIV-1 infection. We took advantage of Ghost-CXCR4 (X4) cell line that could stably express HIV-1 receptor CD4 and co-receptor CXCR4. It contains a GFP reporter gene under the control of HIV-2 LTR promoter [33]. Upon HIV-1 infection, the production of Tat drives the expression of GFP, thus the intensity of GFP could be used as the indication of viral replication. Without infection, only basal level of GFP was expressed (Fig. 2a); while following HIV-1 infection, the production of GFP was greatly increased (Fig. 2b, c). Interestingly, in the ABIN1 knockdown Ghost-X4 cells, the total number of GFP positive cells showed no significant increase (Fig. 2b, c), suggesting that ABIN1 does not affect the binding or entry process during viral infection. However, in ABIN1 depleted cells the mean fluorescence intensity (MFI) and median of GFP intensity show significant increase (Fig. 2f, g) compared to the negative control cells transfected with siNC. The increased MFI represents that the overall LTR promoter activity is up-regulated; while higher MFI indicates that among the infected cells there are more populations which exhibit higher replication rate. To show the differences more clearly, we squared and amplified this part of Fig. 2d to make Fig. 2e, and quantitated the figures to make Fig. 2f, g. The knockdown efficiency of ABIN1 siRNAs (siABIN1-1, siABIN1-2) were shown by Western blots (Fig. 2h). To assure the viral infection to be single-rounded and avoid the interference of newly produced viruses, the cells were collected and analyzed 2 days post infection. Collectively, these data indicate that intrinsic ABIN1 suppresses HIV-1 replication after viral entry into the cells and inhibits the LTR promoter activity.

**Fig. 3:**
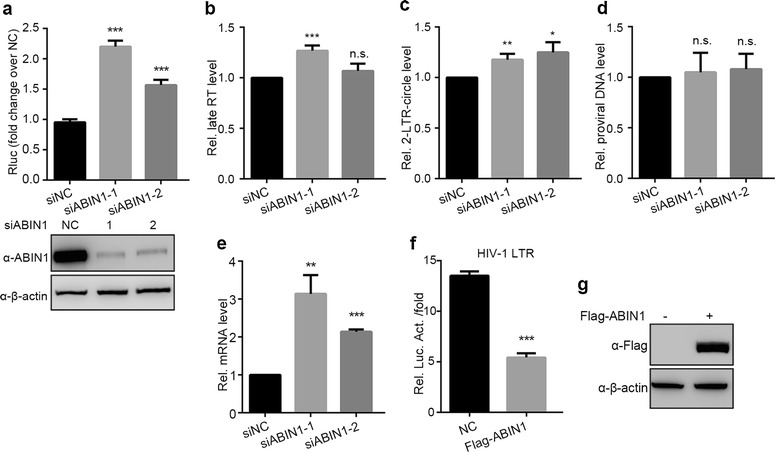
ABIN1 depletion stimulates the activity of HIV-1 promoter (HeLa cells). **a**–**e** HeLa cells were transfected with siNC, siABIN1-1, or siABIN1-2 for 36 h, then cells were challenged with VSV-G pseudotyped luciferase reporter HIV-1(Luc). Cells were harvested at the indicated time points post infection for luciferase activity analysis, DNA or RNA extraction. **a** Luciferase activities of the infected cells were monitored at 48 hpi (*upper panel*) and ABIN1 knockdown efficiency was determined by Western Blots as the representative (*lower panel*). **b**–**d** Total DNA of the infected cells were extracted and quantified for subsequent real-time PCR at **b** 12 hpi for late RT levels, **c** 24 hpi 2-LTR-circle levels, **d** 48 hpi proviral DNA levels. **e** Total RNA was extracted at 48 hpi, and used for quantitation of HIV-1 mRNA. **f** The effect of overexpressed ABIN1 on LTR promoter activity were determined by luciferase assays after co-transfection of Flag-ABIN1 expressing plasmid or vector control together with pNL4-3.Luc.R-.E-, and pRL-TK in HEK-293T cells for 24 h. **g** Expression of Flag-ABIN1 was determined by Western Blots. The real-time PCR analysis of DNA and RNA extract were normalized to cellular β-globin and GAPDH, respectively. The primer pairs used were as described in “Methods”. Data are represented as mean ± SD of triplicate samples and are representative of at least three independent experiments. *p* values were calculated based on unpaired *t* test and significant changes relative to siNC or NC indicated. *p < 0.05; **p < 0.01; ***p < 0.001, *ns* not significant

To further explore the phases of the HIV-1 life cycle where ABIN1 functions to inhibit viral replication after entry, we transiently transfected HeLa cells with ABIN1 siRNAs (siABIN1-1, siABIN1-2 or siNC) to make ABIN1 knockdown and control cells, and then transfected them with VSV-G pseudotyped single-cycle HIV-1(Luc). As an ideal model for this research, HIV-1(Luc) lacks envelop so that its infection does not produce infectious viruses. In addition, it encodes a firefly luciferase in the locus of Nef, making it convenient to measure the replication level [34]. As described in “Methods”, various products that appear at different phases of the viral life cycle have been analyzed and quantified, including late RT, 2-LTR-circles, integrated proviral DNA and the mRNA transcribed from the provirus. In consistent with our experiments conducted in Jurkat T cells and primary human CD4+ T lymphocytes, ABIN1 knockdown in HeLa cells also leads to increased viral replication compared to siNC cells, to about 1.5-fold to 2.2-fold measured by luciferase assay (Fig. 3a, the upper panel). The depletion of ABIN1 protein was confirmed by Western blot analysis (Fig. 3a, the lower panel). We found that the product of late RT, 2-LTR-circles and integrated proviral DNA, measured at 12, 24 and 48 h post infection respectively, neither displayed significant difference between control and ABIN1 knockdown HeLa cells (Fig. 3b, d). However, the mRNA level of HIV-1 post-integration transcription in ABIN1 knockdown cells increased to twofold to threefold relative to the negative control cells (Fig. 3e). Furthermore, when ABIN1 was cotransfected with proviral plasmid pNL4-3.Luc.R-.E- and reference plasmid pRL-TK in HEK-293T cells, significant reduction of HIV-1 replication has been observed (evaluated by luciferase assay, Fig. 3f). ABIN1 expression was confirmed by Western blot with β-actin as sample loading control (Fig. 3g). These results indicate that ABIN1 does act on the transcription step of integrated HIV-1 proviral genome to suppress the LTR promoter activity but not on the earlier steps of HIV-1 life cycle, since depletion of ABIN1 only led to augmented of HIV-1 mRNA but not late RT products, 2-LTR-circles and integrated proviral genome (Fig. 3). Late RT products indicate the overall reverse transcription occurs immediately after viral infection. 2-LTR circles are non-productive forms of cDNAs derived from the reverse transcription products, which is also associated with viral infection progression [35, 36].

### Identification of HIV-1 Tat as the key factor targeted by ABIN1 via its ubiquitin binding property

In previous studies, ABIN1 has been suggested to function as an ubiquitin sensor to restrict cell death and sustains embryonic development through its polyubiquitin chain binding capacity [2]. In order to elucidate the molecular mechanism that ABIN1 inhibits HIV-1 replication, we tested whether ABIN1 had any effect on the ubiquitination of HIV-1 viral proteins and whether the inhibitory role of ABIN1 on HIV-1 replication would depend on its ubiquitin sensing activity. We targeted three HIV-1 encoded proteins that have been reported to be essential for HIV-1 replication at different stages and can be ubiquitinated, including Tat, Rev and Gag. Accessory proteins Vif, Vpr, Vpu and Nef were excluded because they are not directly involved in the viral replication cycle.

**Fig. 4:**
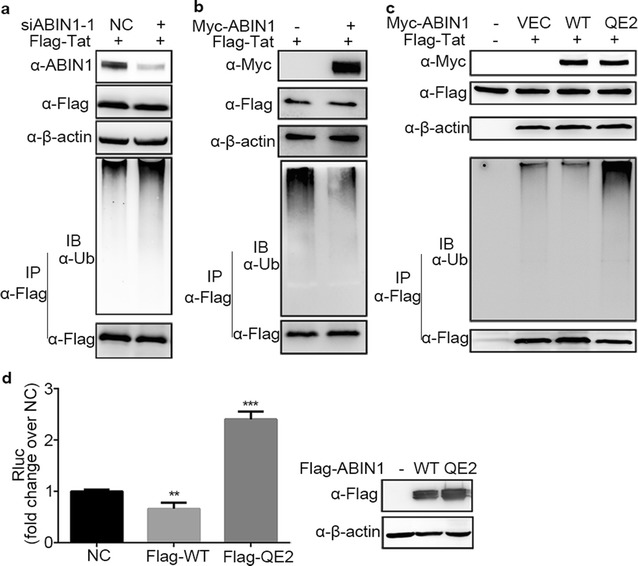
ABIN1 suppresses HIV-1 Tat ubiquitination via its ubiquitin binding property. **a** HEK-293T cells were transfected with siRNAs targeting ABIN1 (siABIN1-1, siABIN1-2) or siNC for 24 h, then a second round transfection of Flag-Tat expressing constructs was performed for another 24 h, and cells were then harvested and assayed as described in “Methods”. **b** After transfection with Myc-ABIN1 and Flag-Tat expressing plasmids for 24 h  as described in “Methods”, the ubiquitination of Tat in HEK-293T cells was analyzed by Flag IP under denaturing conditions as in (**a**). **c** HEK-293T cells were co-transfected with plasmids encoding Myc tagged wild-type ABIN1 or ABIN1-QE2 mutant and Flag-Tat for 24 h, cells were then harvested and analyzed as in (**a**). **d** The effect of overexpressed ABIN1 and QE2 mutant were determined by luciferase assays after HIV-1(Luc) infection following ABIN1, QE2 or vector control (NC) transfection in HeLa cells. The *panel below* indicates the expression of ABIN1 or its mutant. β-actin was detected as sample loading control. Data are represented as mean ± SD of triplicate samples, all data and Western Blots are representative of at least three independent experiments. *p* values were calculated based on unpaired *t* test and significant changes relative to NC indicated. *p < 0.05; **p < 0.01; ***p < 0.001

Tat, Rev or Gag were transfected into ABIN1 knockdown and control HEK-293T cells, respectively. To avoid the interference caused by their interacting proteins that were also ubiquitinated, Tat, Rev and Gag proteins were immunoprecipitated under denaturing conditions and were then blotted with ubiquitin antibody. The results showed that depletion of ABIN1 significantly up-regulated the ubiquitination level of Tat (Fig. 4a) but had no influence on the ubiquitination level of Rev and Gag (data not shown). Moreover, over-expression of ABIN1 in HEK-293T cells caused obvious reduction of Tat ubiquitination (Fig. 4b). Therefore, we concluded that ABIN1 can reduce the ubiquitination level of Tat protein during HIV-1 infection. Considering the previous report that ubiquitination of Tat could stimulate the transcription of HIV-1, we thus speculate that ABIN1 may limit HIV-1 replication by reducing the ubiquitination level of Tat and disturbing the stimulating of HIV-1 transcription.

In order to study whether the ubiquitin chain binding ability of ABIN1 is crucial to its inhibitory role on HIV-1 replication, we utilized an ABIN1 mutant QE2 to abrogate the ubiquitin binding activity of ABIN1. This mutant had two conserved glutamines (Q) mutated to glutamic acid (E) at 464/465 positions (QE2) among the C-terminal UBAN domain of ABIN1 and had been shown previously to lost ubiquitin chain binding ability [2]. The ubiquitination level of Tat was no longer down-regulated when QE2 mutant replaced wild-type ABIN1 to be co-transfected with Tat, conversely, compared to the vector control it was even up-regulated (Fig. 4c). Such dominant negative property has been reported to be shown by mouse ABIN1 mutants that bear QQ_477/478_EE or D485N mutations and lost the ubiquitin binding property [2, 3]. These data indicate that the ubiquitin chain binding property is essential for the inhibitory role of ABIN1. Based on this, we predicted that ABIN1 QE2 mutant would no longer protect cells from HIV-1 infection, instead, it might cause even worse situation for cells.

The result was exactly the same as we predicted. When ABIN1 QE2-transfected HeLa cells were infected with VSV-G pseudotyped HIV-1 luciferase reporter virus HIV-1(Luc), HIV-1 replication increased to around 2.5-fold relative to vector control NC, as measured by luciferase activity (Fig. 4d, upper panel). This up-regulation of viral replication was concordant with the increased ubiquitinated Tat conjugates observed in ABIN1 QE2-transfected HeLa cells (Fig. 4c). While similar amount of wild-type ABIN1 and QE2 mutant were detected by Western Blot (Fig. 4d, lower panel), their effects on host cells turned out to be opposite.

Taken together, our data indicates that ABIN1 limits the stimulation of the transactivation activity of Tat and inhibits HIV-1 transcription by affecting Tat ubiquitination. The ability of ABIN1 to bind ubiquitin chain is essential for its inhibitory effect on HIV-1 replication.

### ABIN1 negatively regulates the K63-linked polyubiquitination of HIV-1 Tat

Previous study showed that Tat undergoes non-proteolytic K63-linked polyubiquitination which mediates the transactivation of HIV-1 promoter [30]. It was also reported that Tat underwent proteolytic K48-linked polyubiquitination which modulates stability and activities of Tat [31]. Thus, our next goal is to determine whether ABIN1 affect the non-proteolytic K63-linked ubiquitination or the proteolytic K48-linked ubiquitination of Tat, or both.

**Fig. 5:**
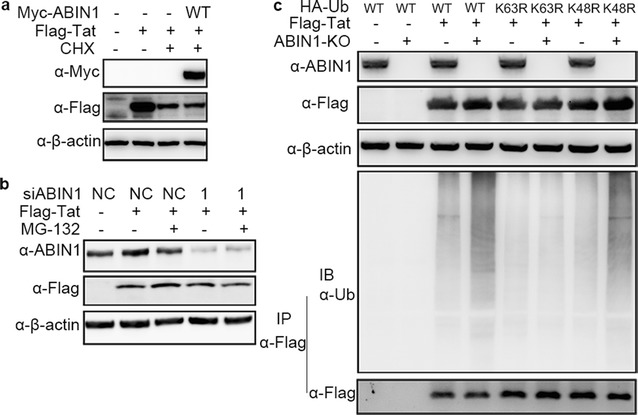
ABIN1 negatively regulates the K63-linked polyubiquitination of HIV-1 Tat. **a** To determine the effect of ABIN1 on the stability of Tat, HEK-293T cells were transfected with Myc-ABIN1 and Flag-Tat encoding plasmids as indicated for 24 h, followed by treatment of CHX at the final concentration of 10 μg/ml for 6 h to arrest cell translation. The levels of Tat protein were detected by Western Blot. **b** To elucidate whether ABIN1 targeting Tat for proteasome-dependent degradation, HEK-293T cells were transfected with siABIN1-1 or siNC for 24 h, followed by a second round transfection of Flag-Tat encoding plasmids, cells were then treated with MG-132 at the final concentration of 10 ng/ml for 2 h to block proteasomal degradation. The accumulation of Flag-Tat were detected by IB. **c** The type of ubiquitination regulated by ABIN1 was explored in HEK-293T or HEK-293T^ABIN1-KO^ cells by immunoprecipitation as in Fig. 4a after transfected with Flag-Tat, together with wild-type HA–Ub, HA–Ub^K63R^ or HA–Ub^K48R^ mutant expression vectors for 24 h. β-actin was detected as sample loading control. The blots were representatives of at least three independent experiments achieving similar results

First, we questioned whether ABIN1 could affect degradation of Tat. HEK-293T cells were treated with CHX to impede Tat synthesis and the degradation of Tat was observed (Fig. 5a). Overexpression of ABIN1 had no effect on protein level of Tat (Fig. 5a), indicating that although ABIN1 affected Tat ubiquitination it could not modulate Tat stability. In addition, the protein level of Tat did not change in ABIN1 knockdown HEK-293T cells either with or without treatment of proteasome catalytic inhibitor MG-132, which blocks proteasome-dependent protein degradation (Fig. 5b). Collectively, these results suggested that the regulation of Tat ubiquitination by ABIN1 did not affect the stability of Tat.

Next, we evaluated whether ubiquitination of Tat affected by ABIN1 was K63-linked. Flag-Tat were transfected into HEK-293T or HEK-293T^ABIN1-KO^ cells generated using CRISPR/Cas9 system, together with wild-type or mutants ubiquitin bearing K63R or K48R mutation. Immunoprecipitation analysis of Tat was conducted under denaturing conditions to assure only covalent Tat conjugates to be precipitated. The results showed that ABIN1 depletion led to augment of Tat ubiquitination when wild-type or K48R ubiquitin were used, but failed to increase Tat ubiquitination when K63R ubiquitin was used (Fig. 5c), supporting the conclusion that ABIN1 inhibits K63-linked but not K48-linked ubiquitination of Tat.

### ABIN1 specifically inhibits Tat ubiquitination at Lys71

**Fig. 6:**
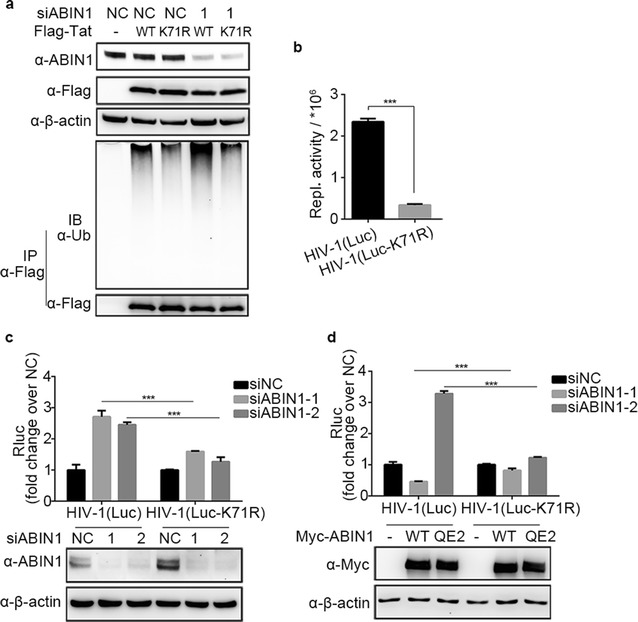
ABIN1 specifically decreases the ubiquitination of Tat at Lys71. **a** HEK-293T cells were treated with siRNAs and plasmids similar as in Fig. 4a as indicated, except that Flag-Tat-K71R mutant was used to assess the ubiquitination of wild-type Tat and Tat^K71R^ mutant in the presence or absence of ABIN1. **b** HeLa cells were infected with HIV-1(Luc) or HIV-1(Luc-K71R) at the same MOI of 0.2, replication activity (Repl. activity) was assessed by luciferase assays 48 hpi to evaluate the importance of Lys71 in Tat. **c**, **d** HEK-293T cells were treated with siRNAs targeting ABIN1 (siABIN1-1, siABIN1-2) or siNC (**c**) or transfected with wild-type ABIN1 or QE2 mutant encoding vectors and challenged with HIV-1(Luc) or HIV-1(Luc-K71R) (**d**), then subjected to luciferase assays 24 hpi to monitor the replication efficiency, and the cell lysates were collected and cleared for detection of knockdown efficiency and overexpression level of ABIN1/mutant. The blots are representatives of at least three independent experiments achieving similar results

Previous studies characterized Lys71 as the major conjugating site of K63-linked polyubiquitin chains on Tat [30]. To further reveal the mechanism that ABIN1 regulates Tat ubiquitination, we investigate whether ABIN1 modulates Tat ubiquitination at Lys71. Wild-type and K71R mutant Tat were transfected into ABIN1 knockdown and control HEK-293T cells respectively. Compared to wild-type, the ubiquitination of K71R mutant was reduced significantly in both ABIN1 knockdown and control HEK-293T cells (Fig. 6a), suggesting that Lys71 is a pivotal ubiquitination site for Tat in vivo. In addition, ABIN1 depletion caused significantly increased ubiquitination of wild-type Tat (Fig. 6a, lane 2 and 4) but not K71R mutant Tat (Fig. 6a, lance 3 and 5), indicating that Lys71 is the key residue of Tat ubiquitination which could be down-regulated by ABIN1.

Next, we further confirmed the functional importance of Tat Lys71 in HIV-1 replication. HeLa cells were challenged with HIV-1(Luc) or HIV-1(Luc-K71R) at the same MOI of 0.2. Results showed that HIV-1(Luc-K71R) almost lost its replication activity compared to HIV-1(Luc) (Fig. 6b), implicating that Lys71 of Tat is critical for HIV-1 replication. Moreover, when Lys71 of Tat was mutated to Arg, the inhibitory effect of ABIN1 on HIV-1 replication was significantly diminished (Fig. 6c, d).

Taken together, we conclude that K63-linked polyubiquitin modification of Tat at Lys71 plays an important role during the process of Tat-stimulated HIV-1 transcription, which is specifically inhibited by cellular ABIN1 protein.

### ABIN1 regulates the ubiquitination of Tat by modulating the distribution of HDM2

**Fig. 7:**
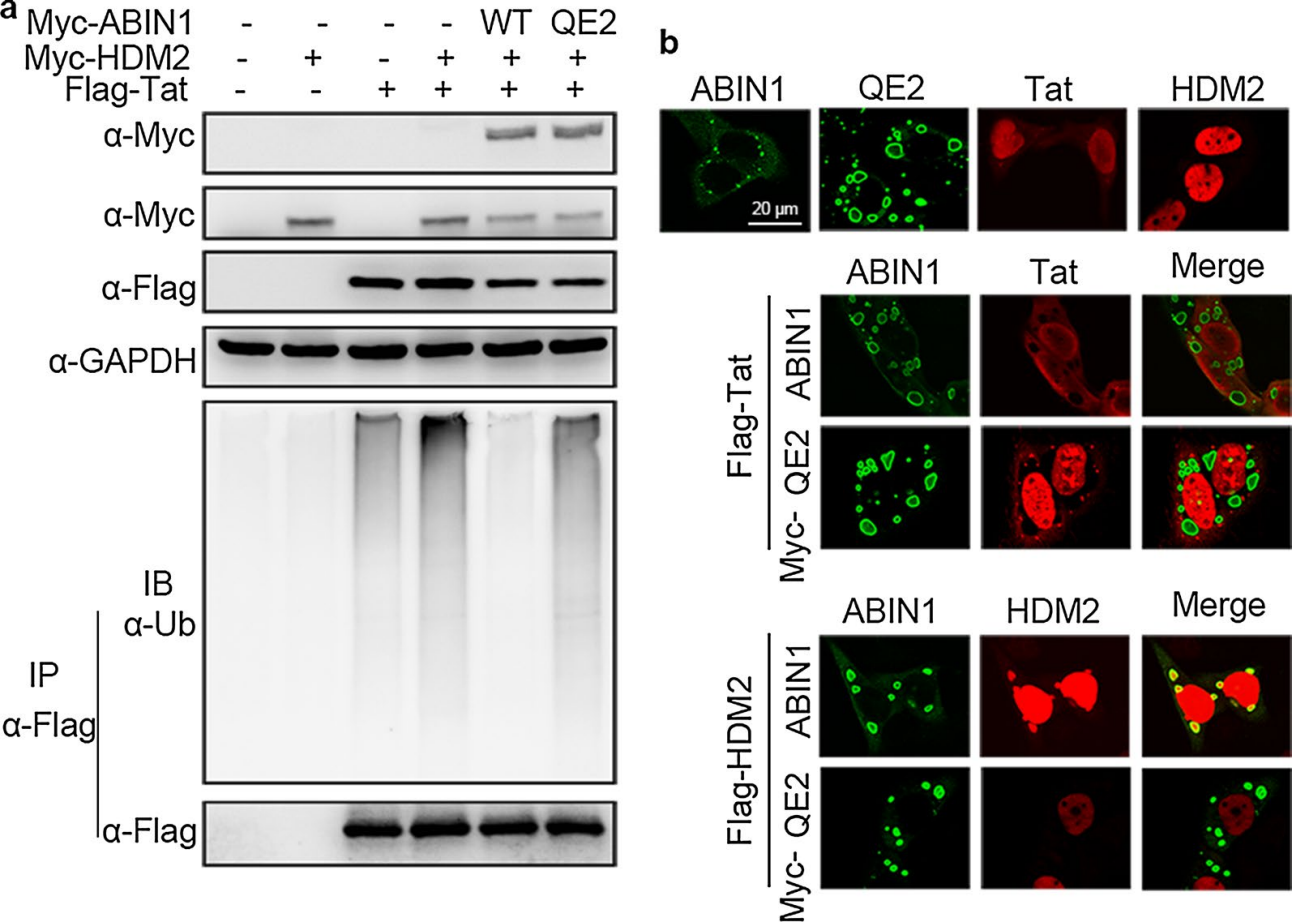
ABIN1 regulates the ubiquitination of Tat by modulating the distribution of HDM2. **a** HEK-293T^ABIN1-KO^ cells were transfected with plasmids encoding Myc-ABIN1 or Myc-QE2, together with Myc-HDM2, Flag-Tat or plasmid vectors as indicated, and subjected to IP as in Fig. 4a. **b** HeLa cells were transfected with expression vectors encoding or Myc-ABIN1, Myc-QE2, Myc-HDM2, Flag-HDM2 or Flag-Tat individually (*upper panel*) or in combination (*middle* and *lower panel*) as indicated for 24 h. Then cells were washed with PBS and harvested for Immunofluorescence analysis. Mouse anti-Myc and rabbit anti-Flag antibodies were used as primary antibodies, FITC-conjugated goat anti-rabbit IgG and Rhodamine-conjugated goat anti-mouse IgG were used to detect the two proteins. The consensus *scale bar* was 20 μm

How ABIN1 regulates Tat ubiquitination remains to be answered. Since ABIN1 can act neither as E3 ligases to ubiquitinate substrates nor as deubiquitin enzymes to disassembled ubiquitin chains, it may functions indirectly on Tat to regulate its ubiquitination. HDM2 emerged from previous report as the E3 ligase of Tat to promote its polyubiquitination [30]. We then tested whether ABIN1 was able to down-regulate HDM2-promoted ubiquitination of Tat. Myc-HDM2 and Flag-Tat were co-transfected into HEK-293T^ABIN1-KO^ cells with Myc-ABIN1 or Myc-ABIN1-QE2 mutant. Flag-Tat was immunoprecipitated under denaturing conditions followed by blotting with ubiquitin antibody. Results showed that wild-type ABIN1 significantly inhibited Tat ubiquitination promoted by HDM2, while ABIN1 QE2 mutant could not (Fig. 7a), supporting the hypothesis that ABIN1 inhibits HDM2-promoted Tat ubiquitination.

More evidence came from our attempt to observe the subcellular localization of ABIN1, Tat and HDM2 proteins. Sparked by the fact that the inhibitory effect of ABIN1 on Tat ubiquitination depends on its ubiquitin binding capacity, we speculated that ABIN1 might bind to HDM2 with ubiquitin as mediator, and thus preventing ubiquitination of Tat by HDM2. Immunofluorescence assays revealed the co-localization of ABIN1 and HDM2 (Fig. 7b). Consistent with their functions, Tat and HDM2 were mainly localized in the nucleus; while ABIN1 were dominantly distributed in the cytoplasm, in the form of non-identified aggregation or vesicles (Fig. 7b, upper panel). When Tat was co-expressed with ABIN1, ABIN1 QE2 or HDM2, the distribution of each protein remained unchanged (Fig. 7b, middle panel). However, ABIN1, but not QE2 mutant, was able to redistribute HDM2 by “pulling” it out of the nucleus to co-localize with ABIN1 in the aggregates (Fig. 7b, lower panel). Collectively, our results support the hypothesis that ABIN1 inhibits Tat ubiquitination by changing the distribution of HDM2, the specific E3 ligase of Tat, via its ubiquitin binding ability.

## Discussion

Hosts have evolved a variety of strategies to recognize and restrict the invading viruses, limiting the damage to survive virus infection. Upon viral invasion, host cells recognize the pathogen-associated molecular patterns (PAMPs) of the viruses by pattern recognition receptors (PRRs), and downstream immune signaling is then activated, finally inducing expression of cellular chemokines and interferons to eliminate the viruses or stimulate further antiviral adaptive immunity response. On the other hand, viruses attempt to protect themselves by all means such as disturbing virus recognition, interrupting immune signaling, and destroying antiviral factors. In a previous report by Gupta K *et at*, the protein level of ABIN1 was shown to be extremely low in resting CD4+ T cells and significantly elevated upon activation by phytohemagglutinin (PHA) [18], while HIV-1 efficiently infects activated CD4+ T cells rather than resting CD4+ T cells. Consistently, our results showed that depletion of ABIN1 in primary CD4+ cells led to promoted HIV-1 replication. In addition, HIV-1 infection had an influence on ABIN1 expression. The mRNA level of ABIN1 was slightly decreased upon acute HIV-1 infection but significantly increased at 72 h post infection, suggesting that ABIN1 may undergo successively regulation by both virus and host. Initially, ABIN1 was suppressed in acute responses to viral infection; some time later it was motivated in response to activated host immune response (Fig. 1b, c). Moreover, recent study by Li et al. [20] suggested that ABIN1 could inhibit the activation of HIV-1 latency. Taken together, we believe that ABIN1 is intimately involved in cellular response to HIV-1 infection and playing an important role in host resistance to HIV-1 replication under natural infection. Therefore, the ABIN1-mediated interaction of HIV-1 and host cells appears to follow the old saying: as vice rises one foot, virtues rise toil.

Although ABIN1 has been previously reported to be involved in HIV-1 replication, both the effects of ABIN1 exerting on virus as well as the underlined molecular mechanism remains ambiguous. In this study, we proved that ABIN1 suppresses HIV-1 replication in primary target cells, the CD4+ T lymphocytes (Fig. 1b), pushing forward the previous study in the transformed HeLa cell line [18]. We also explored the molecular mechanism that ABIN1 negatively regulates HIV-1 replication, which has not been addressed before. Importantly, we determined that ABIN1 inactivates the LTR transcription of the HIV-1 genome at the transcriptional elongation step by regulating the ubiquitination of Tat, a crucial factor for HIV-1 LTR transcription.

To further explore the implications between ABIN1 and Tat, our research proceeds in three lines. The first is to determine the essential domains in ABIN1 for its anti-HIV function. Among those well-known proteins that contain UBAN domains, including ABIN1, NEMO/IKK γ and Optineurin (OPTN), ubiquitin chain binding domains play important roles as a bridge to bring together one ubiquitinated protein with the other regulatory proteins for their function. ABIN1 targets A20 to the K63-linked ubiquitinated NEMO, leading to its deubiquitination and resulting in inhibition of NF-κB signaling [1, 10, 11]. The binding of K63-linked polyubiquitin chains to NEMO is important for the activation of IKK complex and NF-κB signaling [37, 38]. OPTN brings the ubiquitinated proteins to the autophagosomes, leading to the autophagic clearance of them [39]. Collectively, these studies indicate that the polyubiquitin chain binding domain would serve as adaptor and play essential roles during signaling transduction. Therefore, we mutated the conserved glutamines (Q) at 464 and 465 to glutamic acids (E) to abrogate the polyubiquitin chain binding capacity of ABIN1. As expected, we observed that loss of the polyubiquitin chain binding activity results in even increased HIV-1 replication that is in contrary to ABIN1 wild-type (Fig. 4d), demonstrating the essential role of C-terminal UBAN domain of ABIN1 and the dominant negative effect of QE2 mutant. Such dominant negative effect of QE2 mutant was also observed when ABIN1 functions to inhibit the programmed cell death (PCD) induced by tumor necrosis factor α (TNF-α) [2], as well as when ABIN1 functions to prevent autoimmunity [3].

The second line is to determine the ubiquitination type of Tat regulated by ABIN1. Since the bipartite UBAN domain specifically binds K63-linked polyubiquitin chain [40], we focused on such kind of ubiquitin conjugation type. With employment of ubiquitin mutants K63R and K48R as well as proteasome inhibitor MG132, we showed that Tat was not targeted for proteasomal degradation via K48-linked ubiquitination and ABIN1 could regulate K63-linked but not K48-linked ubiquitination of Tat.

The third line is to figure out the key regulatory sites on Tat. Considering that it is the ubiquitination level to be regulated on Tat, we decide to choose ubiquitin-conjugation sites of Tat as candidate. Tat Lys71 has been reported to be the key site for K63-linked polyubiquitination of Tat [30]. In our research, it also turned out to be the key site for Tat ubiquitination regulated by ABIN1.

In order to further explore the molecular mechanism employed by ABIN1 to regulate HIV-1 Tat ubiquitination, we proposed two possible explanation that ABIN1 possibly changes the ubiquitination state of Tat. One is ABIN1 might act as an inhibitor to prevent approaching of Tat to its E3 ligase HDM2, thus abrogating the ubiquitination process. Alternatively, it might function as an adaptor between Tat and a deubiquitinase, where the UBAN domain binds ubiquitinated Tat and other domains binds the deubiquitinase. In our study, ABIN1 was shown to reduce HDM2-catalyzed Tat ubiquitination and co-localization of ABIN1 with HDM2 without involvement of Tat, implying the validity of the former mechanism. A previous study has proved that ubiquitination have no influence on Tat localization [31], thus it is not surprising that ABIN1 has no influence on Tat localization in our experiments (Fig. 7b). In addition, ABIN1 was reported to reside dominantly in the cytoplasm, but it shuttles between nucleus and cytoplasm. We also conducted nucleocytoplasmic separation experiment to confirm the expression of ABIN1 in the nucleus (data not shown). Thus, ABIN1 possibly serves as a shuttle taking HDM2 out from the nucleus, or detains HDM2 in the cytoplasm by preventing HDM2 entering nucleus. In-depth work is required to further solve this question.

People may argue that the inhibitory role of ABIN1 on HIV transcription was due to the inhibitory role of ABIN1 during NF-κB signaling. However, the functions of ABIN1, ABIN2 and ABIN3 was suggested to be redundant as NF-κB inhibitors [1] and ABIN1 has only minimal effect on the basal level of NF-κB activity [1, 2], therefore, we exclude the possibility that ABIN1 inhibits HIV-1 transcription by regulating NF-κB activity by using ABIN1 knockdown or knockout cell lines in most of our studies.

## Conclusions

In summary, our study showed that intrinsic ABIN1 cripples HIV-1 mRNA transcription by decreasing the K63-linked ubiquitination of Tat at the site of Lys71, which is critical for its transactivator function. On one side, such inhibition may help host cells survive from viral infection by limiting their replication; on the other side, it also helps the viruses maintain the latent viral reservoir since the viruses will not be eradicated until the infected cells die. In this point, ABIN1 could make a new target both in the “shock and kill” therapy and gene therapy. Together, our study provides new insights into the host-HIV-1 interaction during viral infection and may offer a potential target for curing HIV-1 infection.

## Methods

### Cell culture and reagents

HEK-293T, HeLa and Jurkat cells were obtained from American Tissue Culture Collection (ATCC), Ghost-CXCR4 (X4) cells were from AIDS Reagents and Reference Program of the NIH. HEK-293T, HEK-293T^ABIN1-KO^, HeLa and Ghost-X4 cells were cultured in Dulbecco’s modified Eagle’s medium (DMEM). Jurkat cells were cultured in RPMI 1640 medium. Culture media were supplemented with 10% fetal bovine serum (FBS), penicillin (100 U/ml) and streptomycin (100 μg/ml), while for Ghost-X4 cells, G418 (500 μg/ml), hygromycin (100 μg/ml) and puromycin (1 μg/ml) were added additionally. HEK-293T^ABIN1-KO^ cells were generated using CRISPR/Cas9 system from HEK-293T cells. Briefly, guide RNAs targeting the third exon of ABIN1 on the genome was constructed into pLenti-CRISPR-V2 plasmid [41], and then transfected into HEK-293T cells. 48 hpt, the transfected cells were screened with puromycin (1.5 μg/ml), followed by mono-clonal screening in 96-well plate. The ABIN1 knockout strain was determined by Western Blot and genome sequencing. Primary CD4+ T lymphocytes were isolated and activated as described [42].

Cycloheximide (CHX) and MG-132 were obtained from Sigma. The antibodies used were as follows: mouse antibodies to Flag (Sigma), Myc (Roche) and β-actin (Proteintech), rabbit antibodies to ABIN1 (Proteintech), Tat (Abcam), and HRP-conjugated anti-ubiquitin antibody (ENZO Life Sciences). Rabbit anti-Flag antibody for immunofluorescence was from Invitrogen.

### Plasmid constructs

All the plasmid constructs were generated using standard molecular biological protocols. pLenti-CRISPR-v2 plasmid was a kind gift from Feng Zhang. pNL4-3 and pNL4-3.Luc.R-.E- had been described elsewhere [43, 44]. ABIN1, HDM2 and Tat coding sequences were PCR amplified from human cDNA or pNL4-3 plasmid and subsequently cloned into the expression plasmid pcDNA4/TO (Invitrogen) with an N-terminal Myc tag, or pRK5 (Genetech) and pEF(generated by our lab) with an N-terminal Flag tag. Myc-ABIN1 was constructed into pcDNA4/TO, Flag-ABIN1 and Myc-HDM2 were constructed into pEF and Flag-Tat was inserted into pRK. Plasmids encoding ABIN1 mutant (Myc-QE2, Flag-QE2 and pEGFP-QE2) were generated by site-directed mutations of ABIN1 coding sequence to generate QQ464/465EE mutations. Flag-TatK71R, pNL4-3-TatK71R and pNL4-3.Luc.TatK71R were generated by introducing a Lys to Arg mutation of Tat at 71, using Flag-Tat, pNL4-3 or pNL4-3.Luc.R-.E- as substrates, respectively.

### Production and titer of HIV-1 viruses

The single-cycle replicative pseudotyped HIV-1 luciferase reporter viruses HIV-1(Luc) or HIV-1(Luc-K71R) were produced by co-transfection of 20 μg pNL4-3.Luc.R-.E- or pNL4-3.Luc.TatK71R together with 4 μg VSV-G expressing plasmid into HEK-293T cells using standard calcium phosphate transfection method, culture medium was replaced with fresh medium at 12 h post transfection (hpt), 48 hpt, the supernatants containing viruses were collected and purified with 0.45 μm filter (Millipore), viral aliquots were stored in −80 °C.

The replication competent CXCR4 (X4)-tropic HIV-1 viruses HIV-1(NL4-3), and HIV-1(NL4-3-K71R) mutant were produced by transient transfection of the viral vector pNL4-3 or pNL4-3-TatK71R mutant into HEK-293T cells, the viruses were harvested and stored as above, with a small difference that the viral supernatants were collected at 72 hpt. The viral titers were measured on Ghost-X4 cells by flowcytometry.

### Transfection of siRNAs

To knockdown ABIN1, HEK-293T, HeLa or Ghost-X4 cells were transfected with individual siRNAs (siNC, siABIN1-1 or siABIN1-2) at a concentration of 50 nM using Lipofectamine 2000 (Life Technologies) according to the manufacturer’s protocol. For Jurkat cells (1 × 10^6^ cells each sample) and activated primary CD4+ T lymphocytes (5 × 10^6^ cells each sample), siRNAs were transduced into the cells by electroporation on 4D-Nucleofector (LONZA) using Amaxa SE cell kits (V4XC, LONZA) for Jurkat cells and Amaxa P3 primary cell kits (V4XP, LONZA) for primary CD4+ T lymphocytes, following manufacturer’s instructions. Cells were then infected with viruses 36 h post siRNA transfection. The siRNAs against human ABIN1 were from Ribobio, the targeted sequences are shown below: siABIN1-1 (siG10331113051): 5′-CCATGAAGCAGCAGTATGA-3′, siABIN1-2 (siG10331113109): 5′-CATTCAAAGATGAGGAGAA-3′. The non-targeting control siNC was provided by Ribobio.

### Viral infections with luciferase reporter virus

VSV-G pseudotyped lentiviral supernatants containing HIV-1(Luc) and HIV-1(Luc-K71R) were generated as described above. Before single-cycle infection, the viruses were digested with DNase I (20 U/ml) at 37 °C for 1 h to remove the residual viral DNA in the virions that may interfere with the following detection. 24 h post siRNA transfection, HeLa cells were incubated with HIV-1(Luc) or HIV-1(Luc-K71R) viral stock for 2 h, in the presence of polybrene (8 μg/ml), the supernatants were then discarded and cells were washed with PBS for three times, then fresh medium was added. 48 h post infection (hpi), luciferase activity assays were performed using the Steady-Glo Luciferase Assay System (Promega).

### Mapping to viral life cycle stages

Viral life cycle stages were measured in HeLa cells using VSV-G pseudotyped HIV-1 reporter virus HIV-1(Luc). HeLa cells were transfected with individual siRNAs and infected with HIV-1(Luc) as described above. At the indicated time points post infection, cells were harvested for DNA isolation using DNA extraction kits (TIANGEN) and the concentrations were quantitated using Nanodrop 2000 (Thermo Fisher). Late RT products and 2-LTR-circle DNA were measured as described in [45], using internal PCR primer pairs: MH531 and MH532 for late RT products, MH535 and MH536 for 2-LTR-circle DNA. Proviral DNA content was measured using the primer pair MH535 and SB704 by Alu PCR as described in [46]. DNA levels were normalized to the cellular house-keeping gene β-globin amplified using the primer pair: β-globin-F: 5′-ACACAACTGTGTTCACTAGC-3′ and β-globin-R: 5′-TGGTCTCCTTAAACCTGTCTTG-3′. To quantify HIV-1 mRNA levels, RNA was extracted using TIANGEN RNA extraction kits and quantitated on a Nanodrop 2000 (Thermo Fisher). 2 μg of the total RNA was reverse transcribed into cDNA using oligo (dT)_15_ primer with MLV Reverse Transcriptase kit (Promega), following the manufacturer’s instructions. The mRNA level of HIV-1 was determined by quantitative PCR using primer set amplifying the gag locus. The sequences are gag-F: 5′- ATCAATGAGGAAGCTGCAGAA-3′ and gag-R: 5′- GATAGGTGGATTATGTGTCAT-3′. The mRNA levels were normalized to the cellular house-keeping gene GAPDH using the primer pair, GAPDH-F: 5′CATGAGAAGTATGACAACAGCCT-3′ and GAPDH-R: 5′-AGTCCTTCCACGATACCAAAGT-3′. All quantitative PCRs were performed using the FastStart Universal SYBR Green Master ROX (Roche) on an Applied Biosystems 7300 Fast Real Time PCR system, by SYBR green detector together with a passive reference dye ROX. The amplification parameters was that initial incubation at 95 °C for 10 min, then 40 cycles of 95 °C for 30 s followed by 1 min at 60 °C (2 min for detection of proviral DNA).

### Infection of replication competent HIV-1 viruses

For infection of Ghost-X4, Jurkat cells and primary CD4+ T lymphocytes, polybrene at the final concentration of 8 μg/ml was added in the medium. Then HIV-1(NL4-3) or HIV-1(NL4-3-K71R) viral stocks were added to the culture medium at the indicated MOI, then cells were incubated at 37 °C for 6 h. Unattached viruses were then removed from the culture medium by three times washing with PBS, then warmed fresh culture medium was added to support cell growth. For Jurkat cells and CD4+ T lymphocytes, cells were then divided into three culture dishes for convenient sample collection according to the time point. Cells and supernatants were harvested for further examination.

### Immunoprecipitation (IP) under denaturing conditions and ubiquitination analysis

Transfected HEK-293T or infected HEK-293T^ABIN1-KO^ cells were washed with ice-cold phosphate-buffered saline and lysed in 400 μl (2 × 10^6^ cells) RIPA lysis buffer (25 mM Tris–HCl pH 7.4, 137 mM NaCl, 1% TritonX-100, 1 mM EDTA) supplemented with protease inhibitors (Roche) and PMSF (Sigma) for 30 min on ice. The cell lysate debris were cleared by centrifugation at 4 °C for 10 min at 12,000*g*. Non-covalent protein–protein interactions were dissociated by heating the cell lysates at 95 °C in 1% SDS (m/v) for 10 min and then samples were diluted 10 times in regular RIPA buffer to make the SDS content 0.1%, avoid interference with IP. For Flag-Tat overexpressing HEK-293T cells, the diluted lysates were incubated with anti-Flag M2 Affinity Gel (Sigma) for 4 h at 4 °C. The beads attached with proteins were subsequently washed 4 times with RIPA lysis buffer, and boiled in 2 × SDS sample loading buffer for 5 min to elute the immunoprecipitated proteins.

### Immunofluorescence

For co-localization staining of ABIN1 and Tat, HeLa cells cultured on slides were transfected with ABIN1 and Tat expressing constructs for 24 h, then washed three times with PBS, fixed for 20 min in 4% paraformaldehyde at room temperature, followed by twice wash with ice-cold PBS and permeating with 0.2% TritonX-100 in PBS for 30 min, then blocked with blocking buffer (3% BSA in PBS) for 30 min at room temperature after another twice wash. Primary antibodies were diluted with 1% BSA in PBS and incubated with cells at room temperature for 2 h. After three times wash with PBS to remove the non-associated antibodies, cells were incubated with secondary antibodies conjugated with FITC (Fluorescein) (Thermo Scientific) and Rhodamine (Thermo Scientific) for 1 h at room temperature. Cells were then washed for three times with PBS and slides were mounted with Prolong Diamond Antifade Mountant with DAPI (Thermo Scientific) and viewed on Olympus FV1000 upright confocal microscope, using FV10-ASW 1.7 software to acquire images.

### HIV-1 p24 ELISA

The collected culture supernatants containing HIV-1 particles were lysed in 0.2% TritonX-100 prepared in PBS. And consecutive dilutions were made to accommodate the p24 concentration in the linear range of a standard curve. HIV-1 p24 ELISAs were performed with RETRO-TEK HIV-1 p24 Antigen ELISA kits (ZeptoMetrix), according to the manufacturer’s instructions.

### Flowcytometry analysis

Ghost-X4 cells were collected and washed with PBS, then filter through 300 mesh nylon. The percentage of GFP positive cell and GFP intensity (if necessary) were determined on a CyAn ADP flowcytometry (Beckman Coulter) according to the manufacturer’s instructions.

### Statistical analysis

Statistical analysis was performed using GraphPad Prism software. All results are expressed as mean ± SD of at least three independent experiments (n ≥ 3). The *p* value was calculated using unpaired *t* test. In all tests, *p* < 0.05 was considered statistically significant, marked as follows: *p < 0.05; **p < 0.01; ***p < 0.001.

## Abbreviations

ABIN1: A20-binding inhibitor of NF-κB
HIV-1: human immunodeficiency virus type-1
HCV: hepatitis C virus
NF-κB: nuclear factor kappa-light-chain-enhancer of activated B cells
CD3/4/28: cluster of differentiation 3/4/28
HDM2: MDM2 proto-oncogene
NEMO: NF-κB essential modulator protein
UBD: ubiquitin binding domain
LTR: long terminal repeat
TAR: trans-activation response element
ELISA: enzyme-linked immunosorbent assay
PCR: polymerase chain reaction
CHX: cycloheximide
